# Evaluation of Bone Biomarkers in Renal Osteodystrophy

**DOI:** 10.3390/life14121540

**Published:** 2024-11-25

**Authors:** Alinie Pichone, Carlos Perez Gomes, Carolina Aguiar Moreira, Maria Lucia Fleiuss Farias, Maurilo Leite

**Affiliations:** 1 Division of Nephrology, Hospital Universitário Clementino Fraga Filho, Federal University of Rio de Janeiro, Rua Prof. Rodolpho Paulo Rocco, 255-Cidade Universitária, Rio de Janeiro 21941-617, RJ, Brazil; cperez@hucff.ufrj.br (C.P.G.); mleitejr@hucff.ufrj.br (M.L.J.); 2 Division of Endocrinology, Academic Research Center of Pro Renal Institute, Rua Vicente Machado, 2190, Curitiba 80060-900, PR, Brazil; carolina.aguiar.moreira@gmail.com; 3 Division of Endocrinology, Hospital Universitário Clementino Fraga Filho, Federal University of Rio de Janeiro, Rua Prof. Rodolpho Paulo Rocco, 255-Cidade Universitária, Rio de Janeiro 21941-617, RJ, Brazil; fleiuss@hucff.ufrj.br

**Keywords:** bone biomarkers, bone biopsy, hemodialysis, histomorphometry, mineralization, renal osteodystrophy, turnover

## Abstract

Renal osteodystrophy (ROD) represents histological bone changes in patients with chronic kidney disease and is classified according to turnover and mineralization. This cross-sectional study evaluates several bone biomarkers and their ability to discriminate turnover and mineralization defects in hemodialysis (HD) patients. Bone-specific [BSAP] and total [tAP] alkaline phosphatase, procollagen-1 N-terminal propeptide [P1NP], C-terminal cross-linking telopeptide [CTX], intact [iPTH] and whole [wPTH] parathyroid hormone, sclerostin [SOST], fibroblast growth factor 23 [FGF-23], vitamin D, osteoprotegerin [OPG], and receptor activator of nuclear factor κB ligand [RANKL] were collected before the bone biopsy. Thirty-two patients were evaluated by bone histomorphometry, which identified mineralization defects and low and high turnover in 47%, 50%, and 41% of patients, respectively. Bone biomarkers (tAP, BSAP, CTX, P1NP) and hormones (iPTH, wPTH, and SOST) were capable of identifying low and high turnover (AUC > 0.877 and >0.857, respectively, *p* < 0.001). PTH plus AP had the best accuracy for identifying high turnover. BSAP > 2x, iPTH > 8x, and wPTH > 6x upper limit of normal range identified high turnover. Lower calcium values (Ca < 8.7 mg/dL) were correlated with mineralization defects. On the other hand, FGF-23, OPG, and RANKL did not impact the turnover and mineralization. While bone histomorphometry is not widely available, bone biomarkers such as BSAP, P1NP, PTH, and calcium allow the assessment of turnover and mineralization defects in HD patients. Then, using bone biomarkers may help clinicians define treatments for ROD and osteoporosis and monitor therapeutic response.

## 1. Introduction

According to the joint ASN-ERA-ISN investigation, approximately 850 million people worldwide had chronic kidney disease (CKD) in 2021 [1]. Bone changes appear in the early stages of CKD and progress substantially by the end stage, especially in dialysis patients [2,3,4]. The CKD–mineral bone disorder (CKD–MBD) syndrome manifests as laboratory abnormalities, bone and extra-skeletal changes (vascular calcification). Renal osteodystrophy (ROD) represents the histological changes observed in CKD–MBD and is classified according to turnover, mineralization, and volume (TMV system) [5]. Using this system, ROD can be classified as adynamic bone disease (ABD), osteomalacia (OM), mixed uremic osteodystrophy (MUO), and osteitis fibrosa (OF). Since bone volume can be low, normal, or high in all of them, this parameter is not helpful for ROD diagnosis [6].

Bone turnover consists of resorption and formation on the bone surface. It is a complex process that promotes the consumption or release of some substances, depending on the activity and number of cells (osteoblasts, osteocytes, and osteoclasts), hormones, and other regulators. After osteoclast activation and differentiation, osteoclasts adhere to the mineralized bone surface and remove collagen fragments, such as C-terminal cross-linking telopeptide (CTX), as well as calcium (Ca) and phosphate (P), which are released into the blood. Then, the eroded surface is refilled by osteoblasts responsible for the deposition of osteoid, which releases procollagen-1 N-terminal propeptide (P1NP), derived from collagen, and alkaline phosphatase (AP) to mineralize the osteoid matrix [7]. The remodeling can be stimulated by parathyroid hormone (PTH) and bone formation inhibited by sclerostin (SOST). Both osteocytes and osteoblasts regulate turnover through the production of receptor activator of nuclear factor κB ligand (RANKL), which activates osteoclast and osteoprotegerin (OPG) and, in turn, prevents ligation of RANKL to osteoclast RANK-receptor. Mineralization is slower and longer than turnover [8], depending on hydroxyapatite formation and mineral deposition. So, the mineralization defect could be attributed to low levels of calcium, phosphorus, vitamin D, aluminum deposition, and acidosis [9].

Turnover and mineralization defects can affect bone quantity and quality, increasing bone frailty and fracture risk. Although bone biopsy with double-labeled tetracycline is the gold standard for ROD diagnosis [10], it is invasive, painful, cost- and time-consuming, and depends on specialized centers, both for execution and histomorphometry analysis [11]. Therefore, non-invasive diagnostic methods for diagnosing ROD are currently being explored. The assessment of biomarkers is minimally invasive and less expensive, allowing for the identification of remodeling-related bone alterations and follow-up of bone turnover over time.

The diagnostic capability of bone biomarkers and their potential in CKD–MBD is still a scientific gap. Our study evaluates several bone biomarkers and regulators among the different diagnoses of ROD and their ability to discriminate bone turnover or mineralization defects in hemodialysis (HD) patients.

## 2. Patients and Methods

### 2.1. Patients

We included patients 18 years and older, on hemodialysis for at least 6 months, and who regularly followed up in our CKD–MBD outpatient program. A bone biopsy was indicated for pre-treatment with anti-osteoporotic medications, a discrepancy between parathyroid hormone and alkaline phosphatase, unexplained bone pain or fracture, or before parathyroidectomy. Exclusion criteria were as follows: patients with a fracture or orthopedic surgery in the preceding year; patients with known bone diseases (multiple myeloma or Paget); patients using medications that alter bone metabolism, such as glucocorticoids and anticonvulsants; patients with liver disease, to avoid possible interference in the total alkaline phosphatase value; and patients with diabetes mellitus, who may have bone changes due to the underlying disease. Demographic and clinical parameters were recorded. Dialysis vintage was defined as the time from the first day of dialysis to the bone biopsy. Blood samples for laboratory analysis were collected within 7 days prior to the bone biopsy.

This study was approved by the Ethics and Research Committee of the Hospital Universitario Clementino Fraga Filho. All patients signed an informed consent form.

### 2.2. Laboratory Analysis

In the interdialytic period, fasting blood samples were collected, immediately centrifuged, and stored at −80 °C. Biochemical analysis included serum calcium (reference range [RR]: 8.6–10.3 mg/dL), phosphorus (RR: 2.5–4.5 mg/dL), creatinine (RR: 0.6–1.1 mg/dL), and bicarbonate (RR: 22–26 mmol/L). All these parameters were measured using standard laboratory techniques.

Reference levels and analytical techniques for biomarkers of bone turnover were as follows: C-terminal cross-linking telopeptide (CTX) measured by electrochemiluminometric assay (Cobas E600, Roche Diagnostics, Indianapolis, IN, USA) (RR: women < 0.650 ng/mL, men < 0.850 ng/mL), bone-specific alkaline phosphatase (BSAP) measured by chemiluminescence immunoassay (Liaison autoanalyzer, DiaSorin, Saluggia, Italy) (RR: 4.9–26 µg/L), total alkaline phosphatase (tAP) measured using kinetic colorimetric assay (Cobas E600, Roche Diagnostics, Indianapolis, IN, USA) (RR: 35–104 U/L), and procollagen-1 N-terminal propeptide (P1NP) measured by chemiluminometric assay (Cobas E600, Roche Diagnostics, Indianapolis, IN, USA) (RR: premenopausal women 13.8 to 60.9 µg/L, posmenopausal women 16.3 to 73.9 µg/L, men 13.9 to 85.5 µg/L).

Intact parathyroid hormone (iPTH) was measured using a chemiluminescence assay (Immulite, Siemens, CA, USA) (RR: 10–65 pg/mL); whole PTH (wPTH) was measured by electrochemiluminometric assay (RR: 20–58 pg/mL); 25-hydroxyvitamin D was measured by competitive chemiluminescent immunoassay (Elecsys, Roche, Berlin, Germany) (RR: 30–60 ng/mL); and 1,25 hydroxyvitamin D was measured by tandem mass spectrometry (RR: 18–72 pg/mL). Sclerostin (SOST) was measured using the R&D human SOST Quantikine ELISA kit (DSST00, Minneapolis, MN, USA) (RR 67–300 pg/mL). Fibroblast growth factor 23 (FGF-23 C-terminal) was measured by ELISA (Synlab, Barcelona, Spain) (RR: 26–110 kRU/L). Serum osteoprotegerin (OPG) and receptor activator of nuclear factor κB ligand (RANKL) were measured by ELISA (Biomedica, Vienna, Austria) (median concentration of a healthy population: 2.7 pmol/L and 0.14 pmol/L, respectively).

### 2.3. Bone Biopsy and Histomorphometry

A percutaneous bone biopsy of the anterior iliac crest was performed with a 7.5 mm trephine, 2 cm inferior, and 2 cm posterior to the anterosuperior iliac crest after double-labeling with tetracycline. The protocol included pre-treatment with hydrochloride tetracycline (20 mg/kg/day) for three consecutive days, with an interval of ten days without medication, followed by another three days of tetracycline administration. The biopsy was performed three to five days after the last dose of tetracycline. The samples were fixed in 70% ethanol, and the undecalcified fragment was impregnated in methylmethacrylate according to the standard protocol and analyzed using a microscope (Nikon Labophot II, Tokyo, Japan) equipped with an ultraviolet (UV) high-resolution digital color video camera (Olympus DP71, Center Valley, PA, USA). The image analysis system was a semiautomatic method provided by the Osteomeasure software version 3.3.0.2 (Osteometrics Inc., Atlanta, GA, USA). Toluidine blue was used as a standard stain. Solochrome azurine was used to assess aluminum bone deposition. All analyses were performed by the same investigator, and the histomorphometric parameters were described as suggested by the American Society of Bone and Mineral Research (ASBMR) Histomorphometry Nomenclature Committee [12]. The following static and dynamic parameters were evaluated: cortical thickness (Ct.Th), trabecular bone volume (BV/TV), bone formation rate per unit of bone surface (BFR/BS), osteoid thickness (O.Th), mineralization lag time (Mlt), and osteoid volume (OV/BV).

The reference ranges of static parameters were obtained from healthy Brazilian subjects covering the gender and age range of the study population [13], and dynamic data were defined from previously reported data [14,15]. Bone turnover was classified as normal when BFR/BS was 1.8 to 3.8 mm^3^/cm^2^/year or 0.05 to 0.10 μm^3^/μm^2^/day [11]. Then, turnover was defined as low when BFR/BS was less than 1.8 mm^3^/cm^2^/year and high when it was above 3.8 mm^3^/cm^2^/year. Mineralization defects were identified when O.Th was above 20 μm and Mlt was above 50 days [14]. In line with ASBMR Histomorphometry Nomenclature Committee recommendations, we input 0.3 µm/day for MAR in the biopsies where only single labels or too few double labels existed [16].

ROD was diagnosed based on TMV classification as follows: adynamic bone disease (ABD), when histomorphometry presented low bone turnover only; osteomalacia (OM), when mineralization defect plus low or normal bone turnover was present; mixed uremic osteodystrophy (MUO), when mineralization defect plus high bone turnover was present; and osteitis fibrosa (OF), when there was high bone turnover only.

### 2.4. Statistical Analysis

Qualitative variables were expressed as absolute numbers and percentages, while continuous variables were presented as mean ± standard deviation or median (interquartile interval). An unpaired *t*-test or Mann–Whitney U test was used for two-group comparisons. An analysis of variance (ANOVA) with a Tukey’s post-hoc test or a Kruskal–Wallis test followed by a Dunn test was used to compare three or more groups. A Pearson’s or Spearman’s correlation was used according to the distribution of variables. A receiver operator characteristic (ROC) curve was used to evaluate the abilities of bone biomarkers and hormones to identify turnover, mineralization, or ROD diagnosis. Positive and negative predictive values were calculated based on sensitivity, specificity, and prevalence. Accuracy was calculated using the following formula: specificity + (prevalence × [sensitivity − specificity]). All tests were two-sided, and the significance level was fixed at 0.05. Statistical analyses were performed using IBM SPSS Statistics, version 28 (Chicago, IL, USA).

## 3. Results

Thirty-seven patients with bone biopsy indication were recruited for the study. Thirty-two individuals provided an adequate sample for histomorphometry analysis and completed all the tests between 2018 and 2022. The age range was from 28 to 71 years, and the HD vintage was from 2 to 30 years. No patient had a history of kidney transplant. Forty-seven percent of the patients were white, and the remaining were black or descended. Nineteen patients were females, and eleven were postmenopausal. No patient used a phosphate binder containing aluminum. All patients underwent intermittent HD three times per week by arteriovenous fistula, with adequate dialysis efficiency (Kt/V: 1.28 ± 0.06). Dialysate calcium was 3.0 mEq/L in 72%, 3.5 mEq/L in 25%, and 2.5 mEq/L in 3% of the patients. The mean blood flow was 400 mL per minute, and the dialysate flow was 500 mL/min. None had residual diuresis (assumed to be higher than 100 mL/24 h). The main clinical and laboratory characteristics of the patients are presented in Table 1.

Histomorphometry analysis of the iliac crest core obtained by bone biopsy identified a mineralization defect in 47% of the patients, low turnover in 50%, and high turnover in 41%. None of the patients had bone aluminum deposition. Regarding the ROD diagnosis, three patients (9%) presented normal parameters, seven patients (22%) presented ABD, nine patients (28%) had OM, six (19%) had MUO, and seven (22%) had OF.

### 3.1. Turnover

Clinical, laboratory, and histomorphometry data were classified according to bone turnover, as presented in Table 2.

**Figure 1:**
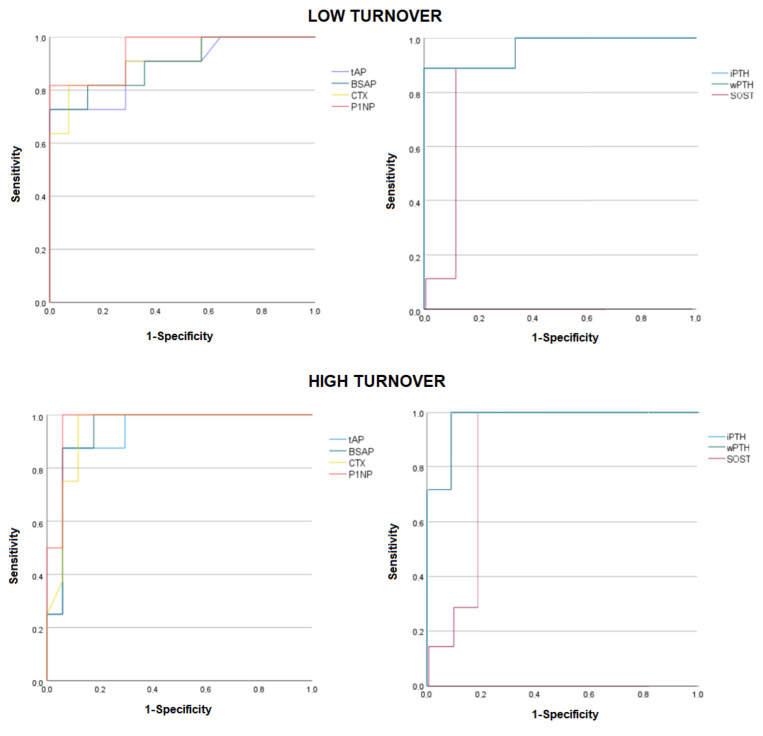
ROC curves of bone biomarkers and hormones for discriminating low from non-low turnover (above) and high from non-high turnover (below). tAP: total alkaline phosphatase; BSAP: bone alkaline phosphatase; iPTH: intact PTH; wPTH: bioactive PTH; CTX: cross-linked C-telopeptide of type I collagen; P1NP: procollagen type 1 N-terminal propeptide; SOST: sclerostin.

To identify patients with low and high turnover using the ROC curve, bone biomarkers (tAP, BSAP, CTX, and P1NP) and hormones (iPTH, wPTH, and SOST) were used to discriminate between low and high turnover, as presented in Figure 1. Combining biomarkers did not increase the diagnostic accuracy when discriminating low turnover from non-low turnover. However, the association of biomarkers (CTX, P1NP, and AP) and PTH improved the AUC when identifying high turnover, even better than the isolated value of PTH. Combining PTH (intact or whole) with alkaline phosphatase (bone or total) was the best parameter to identify high turnover in our study. Cutoffs, predictive values, and accuracy are shown in Table 3.

There were significant correlations among laboratory parameters and between laboratory parameters and dynamic histomorphometry data (Supplementary Table S1). iPTH values exhibited a significant correlation with whole PTH (r = 0.995, *p* < 0.001), with levels of wPTH corresponding to 60% of iPTH. Biomarkers of bone formation (tAP, BSAP, and P1NP) and bone reabsorption (CTX) were positively correlated with PTH (r > 0.750, *p* < 0.001), while SOST showed an inverse correlation with PTH and the biomarkers. The biomarkers and PTH had positive correlations with turnover rates by histomorphometry (r > 0.725, *p* < 0.001), whereas SOST was inversely correlated with BFR/BS (r = −0.696, *p* = 0.003). SOST positively correlated with RANKL (r = 0.577, *p* = 0.012).

### 3.2. Mineralization

Clinical, laboratory, and histomorphometric data according to mineralization are shown in Table 4.

**Figure 2:**
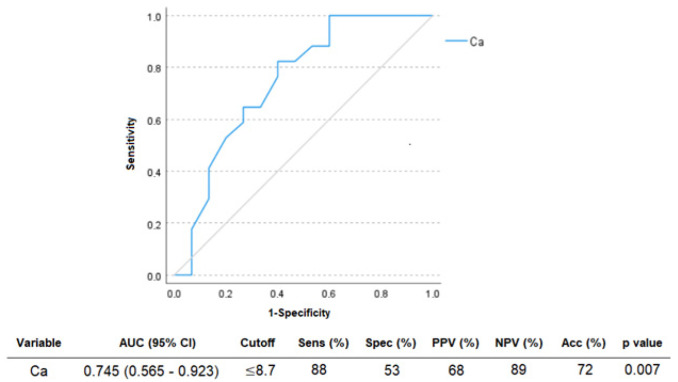
ROC curves of calcium (Ca) to discriminate patients with a defect of mineralization. AUC: area under the ROC curve; CI: confidence interval; Sens: sensitivity; Spec: specificity; PPV: positive predictive value; NPV: negative predictive value; Acc: accuracy.

The ROC curve was used to discriminate between normal and abnormal mineralization. Calcium was used to identify patients with a mineralization defect (AUC 0.745, *p* 0.007). The ROC curve, cutoffs, predictive values, and accuracy are shown in Figure 2.

We observed an inverse correlation between Ca and Mlt (r = −0.466, *p* = 0.038), but there were no correlations among Ca, *p*, bicarbonate, FGF-23, 25(OH), or 1,25(OH)_2_ vitamin D.

## 4. Discussion

Bone biomarkers have been used to assess fracture prediction and monitor osteoporosis in the general population. Patients with CKD present elevated fracture risk, but they are still underdiagnosed and undertreated [17]. Low or high turnover, as well as abnormal mineralization, impact bone quality and frailty. Furthermore, in patients with CKD, bone turnover can determine the choice of osteoporosis treatment [18]. Our study showed that bone resorption and formation biomarkers, in addition to hormones, presented good accuracy for identifying turnover and mineralization defects.

### 4.1. Turnover

The majority of our patients had low turnover, and this prevalence has increased worldwide due to the greater availability of medications to treat hyperparathyroidism, presumably leading to over-treatment [14,19,20,21]. In addition, a high prevalence of mineralization defects was observed in our patients, in agreement with other studies [22,23]. The prevalence of low turnover and mineralization defects has been highly variable in the literature due to the lack of consensus on a definition for normal turnover or mineralization [6,20,24]. In our study, patients with normal turnover had a lower HD vintage, suggesting that patients with longer HD vintage have more exposure to electrolytes and metabolic changes, which predisposed them to bone changes. Furthermore, among histomorphometry structural parameters, patients with high turnover had less Ct.Th, suggesting a catabolic effect of PTH on this compartment [25,26,27].

Consistent with previous reports [19,28], significant differences in the biomarkers of bone formation and resorption, as well as PTH and SOST levels, were observed between patients with low and high turnover. The levels of iPTH and P1NP showed high and comparable accuracies when identifying low bone turnover. In our study, the association of PTH and alkaline phosphatase was the best and most accurate indicator for identifying high turnover, in agreement with Sprague et al. [19]. It is noteworthy that Kidney Disease: Improving Global Outcomes (KDIGO) guidelines suggest that turnover should be evaluated using iPTH and BSAP [29,30].

Interestingly, iPTH was slightly better than wPTH at predicting low turnover. As expected, inactive fragments do not stimulate adenylate cyclase and induce bone resistance to PTH [31]. The influence of inactive fragments detected by the intact PTH assay could also be observed when evaluating high turnover. In fact, a value six times the upper limit of normal (ULN) range for wPTH was able to identify high turnover, whereas a value eight times the ULN for iPTH was required for high turnover identification. Notably, in this focus, iPTH cutoffs were very close to those suggested by KDIGO (2-9x ULN) [29,30].

In the present study, there were positive correlations between bone formation parameters (tAP, BSAP, and P1NP) and a bone resorption parameter (CTX) with intact and whole PTH, in agreement with previous studies [22,28,32]. As expected, SOST showed a negative correlation with BFR/BS since it is a Wnt pathway inhibitor and, consequently, inhibits osteoblastic activity and turnover [33,34].

In hemodialysis patients, FGF-23 levels increase markedly and often reach 1000-fold above the normal range [35], as found in our study. However, there was no difference between the groups regarding bone turnover, similar to RANKL and OPG. This fact can be explained by the production of FGF-23 and RANKL being stimulated by both sclerostin [36,37] and PTH [38,39], predominant in low and high turnover, respectively.

### 4.2. Mineralization

Compared to turnover, biomarkers are less expressive when assessing mineralization, a longer physicochemical process [8]. Mineralization defect was correlated with lower values of calcium. The value close to the lower limit of normality could identify a mineralization defect (AUC > 0.745, *p* = 0.007). Since calcium and phosphorus are minerals that form the hydroxyapatite responsible for bone mineralization, their deficiencies might determine mineralization defects. Similar to our study, Malluche et al. [14] found that patients with mineralization defects had lower calcium, with comparable cutoff values. There was no difference in bicarbonate, and no patients had aluminum deposits, which are known to interfere with mineralization in CKD patients [40].

FGF-23 is a phosphatonin and plays a key role in phosphate homeostasis. In excess, whether due to overproduction or a lack of cleavage of the active molecule, it can cause a mineralization defect due to its potent phosphaturic effect, leading to hypophosphatemia and hindering the formation of hydroxyapatite and mineralization [41]. However, in patients with kidney disease or anuric patients, as in our study, high levels of FGF-23 do not have a phosphaturic action. Nevertheless, they may still contribute to a mineralization defect since they inhibit the activation of 1α hydroxylase, causing decreased levels of active vitamin D and a reduction in the intestinal absorption of calcium and phosphorus [42]. In our study, there was a large variation in FGF-23 levels, ranging from 100- to 1000-fold above the normal range. Furthermore, serum phosphorus levels were high in both groups, and the difference in calcemia was significant, despite similar levels of FGF-23, 25(OH), and 1,25(OH)_2_ vitamin D. To our knowledge, no studies demonstrate the influence of the RANKL/RANK/OPG system on bone mineralization.

### 4.3. Final Considerations

Compared to other studies, our results showed better accuracy of bone biomarkers for identifying turnover, probably because those studies included volunteers, pre-dialysis patients, or a heterogeneous population with regard to ethnicity, countries of origin, treatments, and definitions of normal turnover. Our population was relatively homogeneous, with all the patients at the same stage of kidney disease and undergoing the same dialysis modality. Furthermore, we performed bone biopsy in patients with clinical indications who had high HD vintage and extremely low or high PTH levels. Therefore, our results should not be generalized to all HD patients.

As for clinical practice, since bone histomorphometry is invasive, expensive, and not widely available, our results suggest exploiting bone biomarkers, such as PTH, BSAP, and P1NP, can help identify bone turnover in HD patients. One should be aware of patients with calcium close to the lower limit of normality since they have a high risk of mineralization defects. Low and high turnover, as well as mineralization defects, could harm the bone, decreasing bone mineral density and increasing fracture risk, so it is essential to determine the ROD diagnosis and treat CKD–MBD. In addition, the appropriate use of bone biomarkers allows clinicians to monitor therapeutic responses over time. Further studies are necessary to evaluate the role of FGF-23 and the RANK/RANKL/OPG system in renal osteodystrophy and define if new medications, used to improve bone quality in non-dialysis patients, could influence bone turnover and mineralization in HD patients.

## Figures and Tables

**Table 1:** Main clinical parameters.

Parameters	Results
Age (years)	49.2 ± 10.4
Gender (male)	13 (41%)
BMI (kg/m^2^)	23.8 ± 4.2
HD vintage (months)	177.7 ± 84.1
Causes of CKD	
Hypertension	16 (50%)
Undetermined	7 (32%)
Chronic glomerulonephritis	3 (9%)
SLE	2 (6%)
Eclampsia	2 (6%)
ADPKD	2 (6%)
CKD–MBD therapy	
Phosphate binder	29 (90%)
Calcitriol/Paricalcitol	11 (34%)
Cholecalciferol	10 (31%)
Cinacalcet	11 (34%)

BMI: body mass index; HD: hemodialysis; CKD: chronic kidney disease; SLE: systemic lupus erythematous; ADPKD: autosomal dominant polycystic disease. Results are expressed in absolute number (%) or mean ± SD.

**Table 2:** Clinical, laboratory, and histomorphometry data divided by bone turnover.

	Low Turnover n = 16 (50%)	Normal Turnover n = 3 (9%)	High Turnover n = 13 (41%)	*p* Value
Gender (F)	9 (56%)	2 (66%)	8 (61%)	0.925
White	7 (44%)	1 (33%)	4 (54%)	0.413
Age	49 ± 11.1	53.7 ± 5.7	48.3 ± 10.6	0.732
HD vintage	219.7 ± 72.6	84 ± 68.6	147.7 ± 70.5	0.005 ^a,b^
BMI	22.8 ± 3.1	23.5 ± 5.3	25.2 ± 4.9	0.305
Laboratory				
Ca	8.7 ± 1.6	9.6 ± 0.3	9.5 ± 0.8	0.203
P	5.2 ± 2.2	6.3 ± 2.7	6.1 ± 1.4	0.390
tAP	98 (74, 137)	114 (81, 387)	522 (196, 700)	<0.001 ^b^
BSAP	19 (12, 35)	23 (16, 105)	100 (53, 250)	<0.001 ^b^
iPTH	64 (24, 273)	224 (197, 639)	1300 (820, 2186)	<0.001 ^b^
wPTH	71 (22, 182)	121 (120, 354)	931 (550, 1436)	<0.001 ^b^
CTX	1.7 (0.9, 2.9)	2.7 (1.3, 6.4)	7.7 (3.7, 10.2)	0.001 ^b^
P1NP	411 (231, 587)	543 (499, 3260)	6021 (2473, 10,678)	<0.001 ^b^
SOST	224 (126, 516)	87 (30, 145)	49 (29, 92)	0.023 ^b^
OPG	12.5 (10.9, 14.8)	7.4 (5.8, 9)	10.3 (8.7, 13.6)	0.131
RANKL	0.144 ± 0.060	0.041 ± 0.008	0.098 ± 0.083	0.152
FGF-23	10,880 (1120, 91,990)	66,418 (2256, 130,580)	118,845 (3920, 233,050)	0.502
25(OH)D	28.2 ± 8	28.3 ± 5.8	24.1 ± 1.5	0.388
Histomorphometry				
BV/TV	25.2 ± 2.7	24.5 ± 7.9	25.7 ± 5.1	0.986
Ct.Th	0.845 (0.617, 0.954)	0.885 (0.881, 0.887)	0.288 (0.200, 0.635)	0.035 ^c^
BFR/BS	0.008 (0.005, 0.010)	0.073 (0.059, 0.086)	0.134 (0.130, 0.180)	<0.001 ^b^

F: female; Age (years); HD vintage (months); Ca: calcium (mg/dL); phosphorus (mg/dL); tAP: total alkaline phosphatase (U/L); BSAP: bone alkaline phosphatase (µg/L); iPTH: intact PTH (pg/mL); wPTH: bioactive PTH (pg/mL); CTX: cross-linked C-telopeptide of type I collagen (ng/mL); P1NP: procollagen type 1 N-terminal propeptide (µg/L); SOST: sclerostin (pg/mL); OPG: osteoprotegerin (pmol/L); RANKL: receptor activator of nuclear factor kappa-Β ligand (pmol/L); FGF-23: fibroblast growth factor 23 (kRU/L); 25(OH)D: 25-hydroxy vitamin D (ng/mL); BV/TV: bone volume (%); Ct.Th: cortical thickness (mm); BFR/BS: bone formation rate per unit of bone surface (µm^3^/µm^2^/day). Results are expressed in absolute number (%), mean ± SD, or median (interquartile). An analysis of variance (ANOVA) with a Tukey’s post-hoc test or Kruskal–Wallis test followed by a Dunn test was used to compare three or more groups. ^a^: difference between low and normal turnover; ^b^: difference between low and high turnover; ^c^: difference between normal and high turnover.

**Table 3:** Diagnostic accuracy of laboratory parameters for identifying patients with low bone turnover (above) and high turnover (below).

Variables	AUC (95% CI)	Cut-Off	Sens (%)	Spec (%)	PPV (%)	NPV (%)	Acc (%)	*p* Value
	LOW TURNOVER							
tAP	0.912 (0.813–1.000)	≤113	75	87.5	85	78	81	<0.001
BSAP	0.903 (0.778–0.994)	≤26.3	67	82	79	83	74.5	<0.001
P1NP	0.948 (0.865–1.000)	≤491.6	71	100	100	77.5	85.5	<0.001
CTX	0.909 (0.789–0.997)	≤2.63	71	91	89	76	81	<0.001
SOST	0.877 (0.686–0.982)	>183.4	67	100	100	75	83.5	<0.001
iPTH	0.959 (0.917–1.000)	≤169	75	100	100	70	87.5	<0.001
wPTH	0.939 (0.848–1.000)	≤117	66	100	100	74	83	<0.001
	HIGH TURNOVER							
tAP	0.920 (0.820–0.992)	>186	85	94	91	90	90	<0.001
BSAP	0.944 (0.855–0.996)	>53.1	88	94	91	88	92	<0.001
P1NP	0.971 (0.908–1.000)	>978	100	94	89	100	96	<0.001
CTX	0.945 (0.774–0.997)	>3.38	100	88	80	100	92	<0.001
SOST	0.857 (0.667–0.967)	≤106	100	82	72	100	88	<0.001
iPTH	0.976 (0.925–1.000)	>514	100	94	89	100	96	<0.001
wPTH	0.986 (0.951–1.000)	>357	100	94	89	100	96	<0.001
PTH + CTX	0.993 (0.969–1.000)	above	100	94	89	100	96	<0.001
PTH + P1NP	0.993 (0.969–1.000)	above	100	94	89	100	96	<0.001
PTH + tAP	1.000	above	100	100	100	100	100	<0.001
PTH + BSAP	1.000	above	100	100	100	100	100	<0.001

AUC: area under the ROC curve; CI: confidence interval; Sens: sensitivity; Spec: specificity; PPV: positive predictive value; NPV: negative predictive value; Acc: accuracy; tAP: total alkaline phosphatase (U/L); BSAP: bone alkaline phosphatase (µg/L); iPTH: intact PTH (pg/mL); wPTH: bioactive PTH (pg/mL); PTH: intact or whole PTH; CTX: cross-linked C-telopeptide of type I collagen (ng/mL); P1NP: procollagen type 1 N-terminal propeptide (µg/L); SOST: sclerostin (pg/mL).

**Table 4:** Clinical, laboratory, and histomorphometry data divided by mineralization.

	Defect of Mineralization n = 15 (47%)	Normal Mineralization n = 17 (53%)	*p* Value
Gender (F)	10 (67%)	9 (53%)	0.335
Age	49.9 ± 10.5	48.4 ± 10.5	0.773
HD vintage	198.4 ± 64.8	159.5 ± 96.3	0.187
BMI	23.7 ± 4.7	24 ± 3.8	0.432
Laboratory			
Ca	8.5 ± 1.6	9.6 ± 0.7	0.015 *
P	5.2 ± 1.7	6.1 ± 2.1	0.201
tAP	152 (98, 646)	112 (81, 315)	0.350
BSAP	43 (18, 210)	22 (16, 79)	0.247
iPTH	357 (55, 1300)	431 (63, 1042)	0.852
wPTH	182 (27, 837)	189 (59, 568)	0.960
CTX	2.9 (1, 7.3)	2.8 (1.5, 4.9)	0.769
P1NP	547 (248, 6871)	635 (452, 3723)	0.810
SOST	100.9 (34, 406)	106.4 (38, 233)	0.999
OPG	10.9 (9.9, 15.8)	11.7 (7.9, 12.9)	0.515
RANKL	0.141 ± 0.070	0.114 ± 0.085	0.888
FGF-23	1692 (1116, 127,255)	11,890 (7404, 113,615)	0.247
25 (OH)D	24.2 ± 9.5	28.6 ± 6.1	0.119
1,25 (OH)D_2_	10 (5, 14)	10 (5, 21)	0.503
Bicarbonate	23.7 ± 6.9	23.9 ± 3.8	0.537
Histomorphometry			
BV/TV	25.6 ± 12.2	24.9 ± 10.9	0.883
Ct.Th	0.650 (0.186, 0.834)	0.754 (0.243, 0.890)	0.536
OV/BV	14.9 (4.7, 31.9)	2.7 (1.4, 7.4)	<0.001 *
O.Th	20 (15.3, 31.2)	12.3 (8.5, 13.6)	0.013 *
Mlt	286 (145, 579)	34 (18, 44)	<0.001 *

F: female; Age (years); HD vintage (months); Ca: calcium (mg/dL); P: phosphorus (mg/dL); tAP: total alkaline phosphatase (U/L); BSAP: bone alkaline phosphatase (µg/L); iPTH: intact PTH (pg/mL); wPTH: bioactive PTH (pg/mL); CTX: cross-linked C-telopeptide of type I collagen (ng/mL); P1NP: procollagen type 1 N-terminal propeptide (µg/L); SOST: sclerostin (pg/mL); OPG: osteoprotegerin (pmol/L); RANKL: receptor activator of nuclear factor kappa-Β ligand (pmol/L); FGF-23: fibroblast growth factor 23 (kRU/L); 25(OH)D: 25-hydroxy vitamin D (ng/mL); BV/TV: bone volume (%); Ct.Th: cortical thickness (mm); OV/BV: osteoid volume (%); O.Th: osteoid thickness (µm); Mlt: mineralization lag time (day). An unpaired *t*-test or Mann–Whitney U test was used to compare normal mineralization and mineralization defects. Results are expressed in absolute number (%), mean ± SD, or median (interquartile). * *p* < 0.05.

## Data Availability

The original contributions presented in the study are included in the article/Supplementary Material; further inquiries can be directed to the corresponding author.

## Supplementary Materials

The following supporting information can be downloaded at: https://www.mdpi.com/article/10.3390/life14121540/s1, Table S1: Correlation between laboratory and histomorphometric parameters.
