# Supplementary material for: Evaluation of Bone Biomarkers in Renal Osteodystrophy
Source: Life (Basel). 2024 Nov 25;14(12):1540. doi: 10.3390/life14121540 (PMC11679507; doi:10.3390/life14121540)
Supplement: Supplementary file 1 [file life-14-01540-s001.zip › life-3306722-supplementary.pdf]

SUPPLEMENTARY MATERIAL

Table S1: Correlation between laboratory and histomorphometric parameters

		tAP	BSAP	iPTH	wPTH	CTX	P1NP	SOST
BSAP	rho	**0.969**						
	p	**<0.001**						
iPTH	rho	**0.781**	**0.808**					
	p	**<0.001**	**<0.001**					
wPTH	rho	**0.801**	**0.816**	**0.995**				
	p	**<0.001**	**<0.001**	**<0.001**				
CTX	rho	**0.809**	**0.816**	**0.818**	**0.813**			
	p	**<0.001**	**<0.001**	**<0.001**	**<0.001**			
P1NP	rho	**0.867**	**0.872**	**0.750**	**0.754**	**0.879**		
	p	**<0.001**	**<0.001**	**<0.001**	**<0.001**	**<0.001**		
SOST	rho	**-0.773**	**-0.752**	**-0.591**	**-0.591**	**-0.493**	**-0.575**	
	p	**<0.001**	**<0.001**	**0,010**	**0.010**	**0.038**	**0.013**	
BFR/BS	rho	**0.743**	**0.725**	**0.862**	**0.856**	**0.756**	**0.734**	**-0.696**
	p	**<0.001**	**<0.001**	**<0.001**	**<0.001**	**<0.001**	**<0.001**	**0.003**

tAP: total alkaline phosphatase, BSAP: bone alkaline phosphatase, iPTH: intact PTH, wPTH: bioactive PTH, CTX: cross-linked C-telopeptide of type I collagen, P1NP: procollagen type 1 N-terminal propeptide, SOST: sclerostin, BFR/BS: Bone Fomation Rate per unit of Bone surface
